# Evaluation of the Speed, Accuracy and Precision of the QuickMIC Rapid Antibiotic Susceptibility Testing Assay With Gram-Negative Bacteria in a Clinical Setting

**DOI:** 10.3389/fcimb.2022.758262

**Published:** 2022-03-23

**Authors:** Christer Malmberg, Jessie Torpner, Jenny Fernberg, Håkan Öhrn, Jonas Ångström, Cecilia Johansson, Thomas Tängdén, Johan Kreuger

**Affiliations:** ^1^ Department of Medical Cell Biology, Uppsala University, Uppsala, Sweden; ^2^ Gradientech AB, Uppsala, Sweden; ^3^ Department of Medical Sciences, Uppsala University, Uppsala, Sweden

**Keywords:** lab-on-a-chip, rapid antibiotic susceptibility testing, AST, sepsis, microfluidics, blood culture

## Abstract

The rapidly changing landscape of antimicrobial resistance poses a challenge for empirical antibiotic therapy in severely ill patients and highlights the need for fast antibiotic susceptibility diagnostics to guide treatment. Traditional methods for antibiotic susceptibility testing (AST) of bacteria such as broth microdilution (BMD) or the disc diffusion method (DDM) are comparatively slow and show high variability. Rapid AST methods under development often trade speed for resolution, sometimes only measuring responses at a single antibiotic concentration. QuickMIC is a recently developed lab-on-a-chip system for rapid AST. Here we evaluate the performance of the QuickMIC method with regard to speed, precision and accuracy in comparison to traditional diagnostic methods. 151 blood cultures of clinical Gram-negative isolates with a high frequency of drug resistance were tested using the QuickMIC system and compared with BMD for 12 antibiotics. To investigate sample turnaround time and method functionality in a clinical setting, another 41 clinical blood culture samples were acquired from the Uppsala University Hospital and analyzed on site in the clinical laboratory with the QuickMIC system, and compared with DDM for 8 antibiotics routinely used in the clinical laboratory. The overall essential agreement between MIC values obtained by QuickMIC and BMD was 83.4%, with an average time to result of 3 h 2 min (SD: 24.8 min) for the QuickMIC method. For the clinical dataset, the categorical agreement between QuickMIC and DDM was 96.8%, whereas essential and categorical agreement against BMD was 91.0% and 96.7%, respectively, and the total turnaround time as compared to routine diagnostics was shown to be reduced by 40% (33 h vs. 55 h). Interexperiment variability was low (average SD: 44.6% from target MIC) compared to the acceptable standard of ±1 log_2_ unit (*i.e.* -50% to +100% deviation from target MIC) in BMD. We conclude that the QuickMIC method can provide rapid and accurate AST, and may be especially valuable in settings with high resistance rates, and for antibiotics where wildtype and antibiotic-resistant bacteria have MIC distributions that are close or overlapping.

## Introduction

Sepsis has recently been recognized as the most common cause of death globally, with an estimated 48.9 million sepsis cases each year, resulting in approximately 11 million deaths (Rudd et al., 2020). Rapid administration of adequate antibiotics is key to efficient treatment of severe bacterial infections that otherwise can lead to life-threatening conditions such as sepsis or septic shock (Ferrer et al., 2014; Nauclér et al., 2020). According to international guidelines for management of sepsis and septic shock, administration of antibiotics should be initiated within 1 hour from recognition of sepsis (Rhodes et al., 2017), and a number of studies show that early administration of effective therapy results in increased clinical as well as financial benefits (Kumar et al., 2006; Gaieski et al., 2010; Ferrer et al., 2014; Zhang et al., 2015). However, the escalating prevalence of multidrug-resistant bacteria worldwide reduces the probability of empirical antibiotic therapy being microbiologically active (Rhodes et al., 2017). Thus, rapid diagnostics of antibiotic susceptibility is becoming increasingly important to avoid treatment failure. In recent years there has been an increasing focus on achieving faster clinical microbiology diagnostics such as rapid bacterial identification (rID) and rapid antibiotic susceptibility testing (rAST). Routinely used AST methods like broth microdilution (BMD) and the disc diffusion method (DDM) can be made faster, both by improved diagnostic logistics but also as a result of updated and improved protocols. Furthermore, EUCAST recently presented an improved protocol including new breakpoints for rapid read-out of DDM (to be used within 4, 6 and 8 hours of plate inoculation), which indicates the possible speed gains from updated protocols (Jonasson et al., 2020). However, the actual benefits of the more rapid, traditional methods developed so far has been questioned (van den Bijllaardt et al., 2017; Chandrasekaran et al., 2018), due to problems with uneven performance and analysis times still being too long.

Notably, low- and middle-income countries are disproportionally burdened by sepsis mortality, much due to the lack of effective healthcare systems, including diagnostic services. Access to effective diagnostics has been argued to be as fundamental as access to antibiotics (Okeke et al., 2011; Bebell and Muiru, 2014; Merrett et al., 2016), but remains problematic in resource-poor settings due to the lack of appropriate infrastructure needed to effectively implement diagnostic-guided therapy. Hopefully addressing this problem are several new, affordable, automated and networked diagnostic systems capable of being operated in an outpatient setting. Several new diagnostic systems with a focus on molecular diagnostics for rID and rapid resistance screening have recently reached the market, and examples include the GenMark ePlex system, Curetis Unyvero and the BioMerieux FilmArray systems (WHO, 2019). However, to our knowledge there are currently no approved AST diagnostic systems which fit the above discussed criteria.

**Figure 1:**
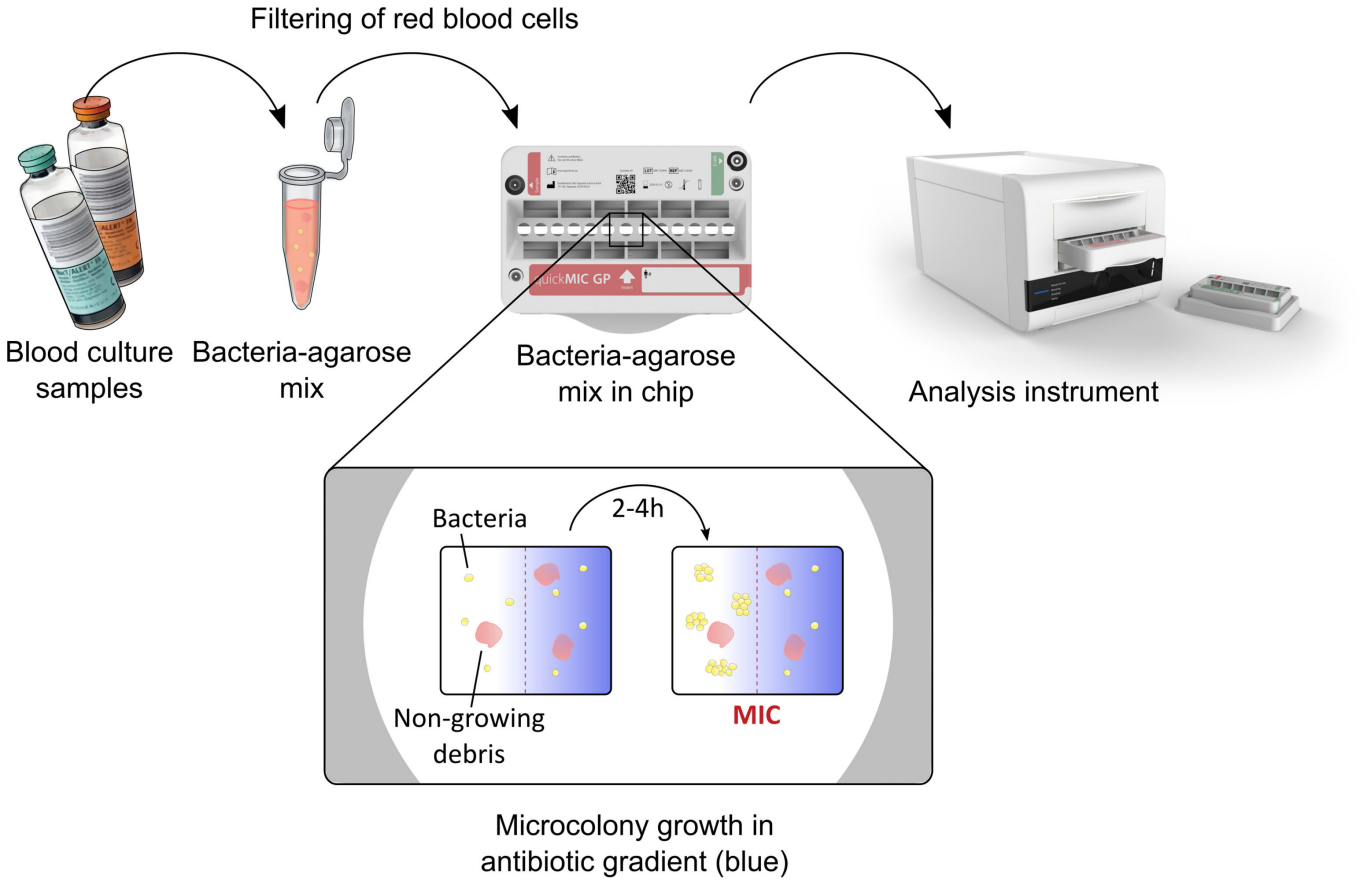
Overview of the QuickMIC system and the sample preparation process. In short, blood culture samples are mixed with a stabilizing agarose matrix, filtered to remove blood cells and injected into the QuickMIC cassette (shown in the middle). The cassette is then inserted into an analysis instrument, where bacterial growth in an array of growth chambers containing polymerized agarose gels is monitored in the presence of linear antibiotic gradients, here represented by blue color. Microcolony growth is evaluated using time-lapse imaging for up to 4 hours, and stable antibiotic inhibition zones are typically detectable after 2-4 h and used to automatically calculate MIC values for the different antibiotics by the QuickMIC software (QM Analyst).

We have previously developed a new microfluidic rAST method (Malmberg et al., 2016; Wistrand-Yuen et al., 2020) where bacterial responses are monitored in precisely controlled antibiotic gradients. We have demonstrated the use of this system for rapid AST of bacteria directly from positive blood cultures, for a single channel prototype system as well as a proof-of-concept multiplex system. In the present study we investigated the performance of a refined system based on our earlier designs, called QuickMIC (**Figure 1**). The ultimate goal is to develop QuickMIC into a rapid, automated and affordable diagnostic solution for AST, with potential to expand access to rapid diagnostics; this new rAST diagnostic system is currently available for research use only (RUO) applications. The aim of this study was to evaluate the performance of the current RUO system with a focus on Gram-negative bacteria and the QuickMIC GN antibiotic panel. The new QuickMIC method was evaluated with respect to accuracy, precision, and time-to-result and also evaluated in a clinical setting. Another goal of the present study was to generate indicators for use in fine-tuning of system performance, and the present study should therefore be considered as a pilot study for further evaluation purposes. Briefly, we show that the QuickMIC system can reduce sample turnaround times in a clinical setting with at least 40% as compared to DDM, and with high categorical agreement (96.8%) to standard testing, and with essential and categorical agreement to reference BMD at 91.0% and 96.7%, respectively.

## Material and Methods

### Handling of Bacterial Isolates for Reference Testing

A total of 151 bacterial isolates (species distribution detailed in **Table 1**) were acquired from several sources, with varying levels of preexisting knowledge of the antibiotic susceptibility profile for each strain. The sources were the EUCAST development laboratory (EDL, Växjö, Sweden), the Antibiotic Research Unit, Uppsala University and Uppsala University Hospital. The isolates were selected to cover distinct antibiotic susceptibility phenotypes, with the aim to include every S/I/R category for each antibiotic on the panel. This selection criteria also minimized the risk for including clonal isolates, since none of the included isolates displayed identical susceptibility pattern. On the day of arrival, the bacterial strains were streaked on agar plates, grown overnight, and harvested into freezing buffer on the next day. The strains were thereafter kept frozen in -70°C for the remainder of the study. All strains were cultivated using Müller-Hinton (MH-II, BBL, Becton Dickinson) agar or cation-adjusted MH-II broth. When preparing spiked cultures for QuickMIC and BMD testing, suspensions of bacteria were achieved by streaking frozen stock on plates and subsequently cultured overnight. The next morning two to four colonies were dissolved in MH-II, after which the suspension was adjusted to 0.5 McFarland. This suspension was either used directly for BMD or diluted and inoculated into BacT/Alert FA Plus bottles (bioMerieux, Marcy l’Etoile, France) together with 10 mL horse blood at an initial concentration of either 25 cfu/mL or 2.5*10^5^ cfu/mL, depending on targeting a short or long incubation. The spiked blood culture bottles were then incubated in a BacT/Alert Virtuo system (bioMerieux) until a positive signal was received, after which the bottle was removed for QuickMIC testing. The timepoints of positive signals as well as the timepoints when the bottles were removed from the BacT/Alert system were recorded.

**Table 1:** Included species and number of strains in the reference panel.

Species		Number of different strains per species	% of all evaluated species
*A. baumannii*		20	13.2
*C. koseri*		8	5.3
*K. aerogenes*		8	5.3
*E. cloacae*		14	9.3
*E. coli*		30	19.9
*K. oxytoca*		5	3.3
*K. pneumoniae*		30	19.9
*P. aeruginosa*		17	11.3
*P. mirabilis*		9	6.0
*S. marcescens*		10	6.6
	**Total:**	151	100

On-scale quality-control (QC) strains were used during the study to repeatedly track the QuickMIC method performance, as per the instructions for use available from the manufacturer (Gradientech AB, Uppsala, Sweden). Results from these continuously performed runs were pooled and intra-laboratory variability calculated for quantification of method repeatability.

### Broth Microdilution AST

Broth microdilution was performed according to ISO 20776-1:2019 (International Organization for Standardization [ISO]). The antibiotics and concentrations used are described in **Table 2**. Briefly, the microplates (Greiner Bio-one, flat bottom PS, product number: 655101) were loaded with antibiotics and inoculated with a bacterial suspension at 0.5 McFarland, yielding a final concentration of ~5*10^5^ CFU/mL; after which the plates were incubated overnight at 37°C. The MIC was read after 16 h, as the lowest drug concentration at which no turbidity could be detected visually. All strains were tested at least twice on separate days, in case of a differing result the strain was retested a third time and the MIC reported as the mode of the three read-outs.

**Table 2:** Antibiotic concentrations used for BMD and QuickMIC AST testing.

Antibiotic (#)	Supplier	Article number	Batch number	Concentration range mg/L (QuickMIC)	Concentration range mg/L (BMD)
Amikacin (AMI)°	Sigma	PHR1860	LRAB1258	1 – 20	0.5-32
Cefepime (CEP)	Sigma	PHR1763	LRAB8503	0.5-10	0.25-16
Ciprofloxacin (CIP)°	Sigma	PHR1044	LRAB3671	0.125-2.5	0.125-4
Colistin (COL)	Sigma	C4461	089M4881V	0.25-5	0.125-8
Cefotaxime (CTA)°	Sigma	Y0000420	4.0	0.25-5	0.0156-8
Ceftazidime/(CTZ)°	Sigma	C0690500	3	0.5-10	0.25-16
Avibactam (CTV)	AmBeed	A169351	A169351-002	4*	4*
Gentamicin sulfate (GEN)°	Sigma	G4918	028M4827V	0.5-10	0.25-16
Meropenem (MER)°	Sigma	PHR1772	LRAB7853	0.5-10	0.25-16
Piperacillin/	Sigma	PHR1805	LRAB7665	2-40/	0.5-64/
Tazobactam (PIT)°	Sigma	68300	BCCD0509	4*	4*
Tigecycline (TIG)	Sigma	Y0001961	1.0	0.06-1.25	0.0156-2
Tobramycin (TOB)°	Sigma	T1783	SLBX8043	0.5-10	0.125-16

#Antibiotic abbreviations according to the EUCAST system for antimicrobial abbreviation, available at www.eucast.org.

*For Ceftazidime/avibactam and Piperacillin/tazobactam combinations, the inhibitor was kept at a fixed concentration of 4 mg/L as per EUCAST guidelines.

°Antibiotic included in routine DDM panel at the clinical microbiology laboratory, Uppsala University Hospital.

### QuickMIC Rapid AST

For QuickMIC testing, samples from positive blood culture bottles were prepared according to the instructions for use available from the manufacturer (Gradientech AB, Uppsala, Sweden). In short, 2-4 mL of blood culture was aspirated using an S-Monovette safety needle (Sarstedt, Nümbrecht, Germany), after which a 10 μL sample loop was used to transfer material to the QuickMIC preparation kit (art. nr: 46-001-10). The preparation kit performs a fixed 1:200 dilution in agarose, followed by a filtering step to remove blood cells using a syringe filter. An aliquot of prepared sample was kept for inoculate control by plating. The sample was then loaded in the QuickMIC test cassette (GN panel, art. nr: 43-001-10) sample port, after which 20 mL of MH-II was injected in the cassette media port. The cassette was inserted into the instrument and the test was started through the control software (QM Analyst v0.93). The QuickMIC RUO (v0.93) system was used for the entirety of this study, in this case consisting of 6 instrument modules connected to a control PC. In the current study, the QuickMIC GN cassettes for Gram-negative bacteria were hand loaded with antibiotics according to the concentrations in **Table 2**, by the following procedure: Each antibiotic well was loaded with 49 μl stock concentration of 10x the concentration indicated in **Table 2**, which was then diluted to final concentration during the MH-II loading step. Each of the 151 strains were tested once using the QuickMIC system.

### AST of Clinical Blood Cultures

A total of 48 clinical samples were collected as per the following protocol. After indication of growth in the BactAlert Virtuo system and indication of Gram-negative bacteria by Gram-stain, 2 mL samples from the blood culture bottles (FA Plus or FN Plus) were provided by the Department of Clinical Microbiology at Uppsala University Hospital. The samples were labelled with a sample code for anonymization, and an aliquot of the sample was streaked on MH-II agar for determination of inoculate concentration, as well as for isolation of the strain. The remaining sample was prepared as described above. Single colonies from the streaked plate were used to create a frozen stock for BMD testing, which was then performed as described above. Afterwards, for all tested strains the bacterial identity (MALDI Biotyper, Bruker Daltoniks), susceptibility category as determined by DDM and data on test dates and times to result were provided by the clinical microbiological laboratory. Only samples where non-facultative Gram-negative pathogens where identified as single species were approved for further data analysis (*n* = 41). After test completion, or at longest 6 months after sample collection, the original blood samples were destroyed and the dataset was de-identified by destruction of the code key. The study protocol for the clinically derived samples was approved by the Swedish Ethical Review Authority (Dnr 2020-03060).

### Data Analysis

A positive control chamber not exposed to antibiotics was included in the QuickMIC cassette, and only cassettes with bacterial growth in the positive control were analyzed. Further, only cassettes where the mean fluidic flow was within ±50% of nominal flow (1 μl/min) in the flow channels at the end of the experiment were accepted as valid runs. Further exclusion criteria included excessive bubble formation and unstable gels in the cassettes as judged by visual inspection. The MIC values generated by the QuickMIC system (QM Analyst v1.0) were compared with BMD values as previously described (Wistrand-Yuen et al., 2020). In short, linear-scale MIC values were right-censored to nearest log2 dilution step, and essential agreement counted as within 1 log2 step of the corresponding BMD result. When the reference method showed results below or above the limit of quantification (LOQ) for the tested method, these were counted as in agreement. For comparison of categorical agreement, susceptible (S) instead of resistant (R) was counted as a very major discrepancy (VMD), R instead of S as a major discrepancy (MD) and S or R instead of increased exposure (I) or vice versa as a minor discrepancy (MiD). The categorization was performed by applying EUCAST clinical breakpoints (version 10.0, 2020, available at www.eucast.org). Data on times to result and turnaround times for each analysis performed were compared using the freely available Jamovi distribution of R (v1.68). For statistical analysis of parameters influencing the result quality, logistic and linear regression was performed using Jamovi. For repeatability analysis, the mean MIC and standard deviation (SD) of MIC of all runs were calculated, and the SD normalized to the target MIC for each tested QC strain and antibiotic.

## Results

### QuickMIC Performance From Spiked Blood Samples

After acquiring a reference strain collection of 151 nonfacultative Gram-negative strains of clinical origin (**Table 1**) we performed rapid AST testing using the QuickMIC system to assess the performance of the novel assay as compared to BMD. Each strain was tested against 12 antibiotics, as detailed in **Table 2**. Using the above described validity criteria to establish if a run was valid or not, 92% of the analyses of the different drug and bacteria combinations were deemed valid (*n* = 1671). MIC values were obtained from the QuickMIC system data analysis algorithm (v1.0), and all MIC values from valid runs were compared to reference MICs from BMD (**Table 3**). During the study, three on-scale quality control (QC) strains (*E. coli* NCTC 13846, *K. pneumoniae* ATCC 700603 and *P. aeruginosa* ATCC 27853) were regularly run with the system to track performance. These replicate runs were performed on separate days (*E. coli n* = 21, *K. pneumoniae n* = 19, *P. aeruginosa n* = 22), and were pooled to measure repeatability of the QuickMIC system.

**Table 3A:** Essential agreement and categorical agreement between QuickMIC and BMD for the reference strains, by tested antibiotic.

*n* =	Antibiotic												
	AMI (100)	CEP (141)	CIP (147)	COL (149)	CTA (137)	CTV(131)	CTZ (144)	GEN (147)	MER (152)	PIT (141)	TIG (145)	TOB (137)	Total (1671)
EA (%)	70.8	84.9	91.0	71.4	86.0	89.3	75.5	91.7	84.1	83.1	87.7	82.0	83.4
CA (%)	94.9	81.7	91.8	92.6	86.9	99.1	72.5	96.9	84.9	78.5	57.6	89.8	87.4
MiD (%)	0.0	14.3	6.1	0.0	4.0	0.0	16.7	0.0	10.5	14.0	0.0	0.0	5.9
MD (%)	0.0	4.0	1.4	0.7	5.1	0.0	8.3	0.8	0.7	5.8	30.3	2.9	3.2
VMD (%)	5.1	0.0	0.7	6.7	4.0	0.9	2.5	2.3	3.9	1.7	12.1	7.3	3.4

**Figure 2:**
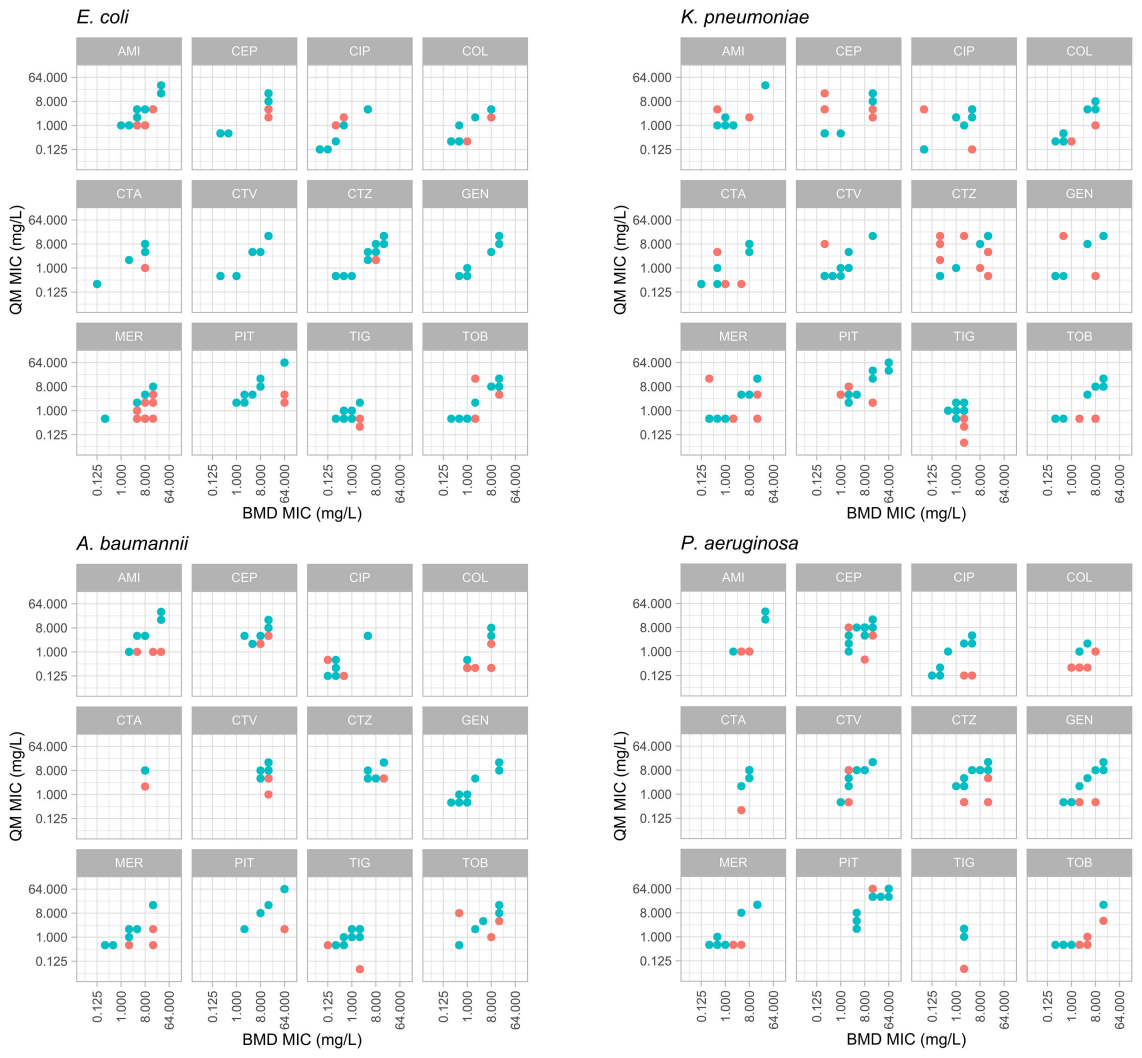
Distribution of MIC value results from the reference BMD method (x-axes) and the QuickMIC (QM) method (y-axes), for 4 common bacterial species encountered in blood stream infections in response to the 12 tested antibiotics. MIC-values obtained by the QuickMIC method where there were essential agreement (EA) with results obtained using BMD are shown in blue and MIC values not in agreement are shown in red. AMI, amikacin; CEP, cefepime; CIP, ciprofloxacin; COL, colistin; CTA, cefotaxime; CTV, ceftazidime/avibactam; CTZ, ceftazidime; GEN, gentamicin; MER, meropenem; PIT, piperacillin/tazobactam; TIG, tigecycline; TOB, tobramycin.

For the antibiotics tested, the essential agreement (EA) between QuickMIC and BMD ranged from 70.8% to 91.7% (mean: 83.4%, *n* = 1671) depending on antibiotic (**Table 3A**) and from 66.7 to 100% depending on species tested (**Table 3B**). The overall categorical agreement (CA) between QuickMIC and BMD for the dataset was 87.4%, ranging from 57.6 to 99.1% depending on antibiotic tested, and from 74.5 to 100% depending on species tested. There was no major difference in performance between non-fermentative bacteria and Enterobacterales. Categorical agreement was only calculated for drug and bacteria combinations with breakpoints as established by EUCAST. The distribution of errors for each antibiotic for the 4 most common Gram-negative bacterial species isolated in blood stream infections can be seen in **Figure 2**. The rates of very major discrepancy against BMD were below 3% for all tested antibiotics except for AMI, COL, CTA, MER and TIG.

**Table 3B:** Essential agreement and categorical agreement between QuickMIC and BMD for the reference strains, by tested species.

n =	Species											
	*E. coli* (352)	*K. pneumoniae* (313)	*P. aeruginosa* (207)	*A. baumannii* (225)	*K. aerogenes* (75)	*E. cloacae* (152)	*K. oxytoca* (54)	*P. mirabilis* (107)	*S. marcescens* (106)	*C. koseri* (80)	Total (fermenters)(1239)	Total (non-fermenters) (432)
EA (%)	88.6	83.9	78.3	80.1	88.0	87.2	100.0	85.8	66.7	77.2	84.9	79.2
CA (%)	86.9	86.9	82.7	88.8	91.4	89.9	100.0	90.7	74.5	81.3	87.6	86.8
MiD (%)	6.6	4.9	10.7	0.9	2.9	7.9	0.0	3.1	4.1	12.5	5.7	6.6
MD (%)	2.0	2.8	0.0	1.9	4.3	0.7	0.0	4.1	16.3	6.3	3.8	0.8
VMD (%)	3.4	4.2	4.0	8.4	1.4	1.4	0.0	2.1	5.1	0.0	2.9	5.8

The QuickMIC system reports individual MIC values from the cassette as soon as they are stabilized, which is called after that the MIC read-out value has fluctuated less than 5% over 30 minutes. The average time to result for the reference strains was 182 min (SD: ± 24.8 min), with a range from 150 to 230 min.

### Method Repeatability

**Figure 3:**
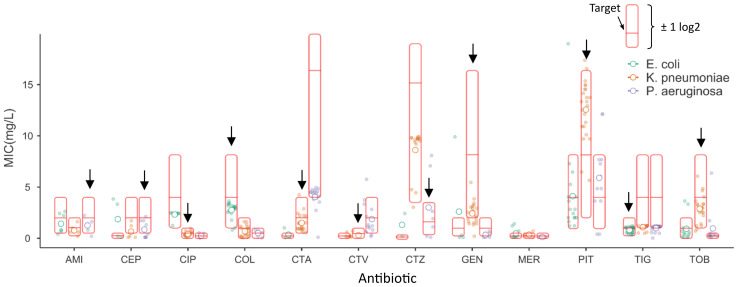
Repeatability per tested antibiotic for the three quality control strains. The linear-scale MIC outputs (i.e. not right-censored for direct comparison with BMD) from the QuickMIC instrument are shown for each bacteria-antibiotic pair relative to the reference MIC value obtained using the BMD method (middle red horizontal line marked “Target”), with the full interval corresponding to essential agreement indicated (by the red box). Open circles correspond to the mean MIC values for each antibiotic, whereas the closed circles correspond to all collected MIC data points for all tested combinations of bacteria and antibiotics. The black arrow identifies the on-scale QC strain for respective antibiotic.

In total, the QC strains were tested on 62 occasions during the 8-month study. The three QC strains were selected to together give an on-scale result for all antibiotics included in the panel (except meropenem, where no on-scale ATCC QC-strain could be found), and were used to track method performance as per the instructions for use of the QuickMIC system. This QC dataset allowed a quantitative determination of method repeatability. The linear-scale MIC results are shown in **Figure 3**, relative to the reference MIC of the strain and the acceptable ±1 log2-step target interval. One strain per antibiotic was used for QC testing (**Figure 3**, black arrow). The method repeatability for each antibiotic, normalized to target MIC for the respective QC strain, is shown in **Table 4**. The average repeatability (SD normalized to target MIC) was 44.6% of the target MIC value, with a range from 13.4 to 150.5%. The highest repeatability was displayed by ceftazidime and the lowest by gentamicin. The acceptable BMD variation corresponds to -50% to +100% variation from the target MIC value on a linear scale, meaning that the results for all antibiotics except for ceftazidime had acceptable variability.

**Table 4:** Repeatability per antibiotic for each panel reference strain. Only on-scale MIC results were used for repeatability calculations.

n =	Antibiotic												
	AMI (5)	CEP (10)	CIP (21)	COL (22)	CTA (24)	CTV (7)	CTZ (8)	GEN (23)	MER (-)	PIT (24)	TIG (25)	TOB (22)	Total (192)
Target MIC	2	2	0.5	4	2	0.5	2	8	–	8	1	4	
Mean QuickMIC linear MIC (right censored)	1.25 (2)	0.86 (1)	0.43 (0.5)	2.76 (4)	1.50 (2)	0.303 (0.5)	3.01 (4)	2.43 (4)	–	12.60 (16)	0.81 (1)	2.88 (4)	
Repeatability (%SD of on-scale MIC)	38.8	40.1	41.2	18.0	42.1	49.4	150.5	13.4	–	35.6	28.6	33.3	44.6
Reference strain*	P	P	K	E	K	K	P	K	–	K	E	K	

*E, E. coli NCTC 13846; K, K. pneumoniae ATCC 700603; P, P. aeruginosa ATCC 27853.

### Performance of the Sample Preparation Process

**Figure 4:**
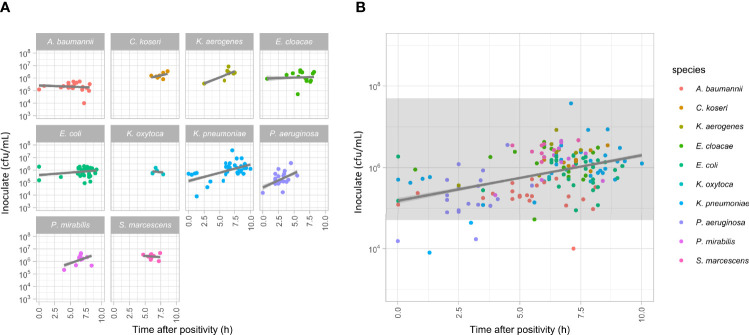
**(A)** Measurement of the amounts of bacteria in blood culture samples plotted against the time from positivity signal of the blood culture bottles to sampling. Linear regression lines (grey lines) are presented for each dataset, no significant correlations were detected. **(B)** Plot showing all the data presented in panel a, together with an indication of the range of sample concentrations compatible with downstream MIC analyses using the QuickMIC system (grey field).

The QuickMIC system is designed to be robust with regards to initial inoculate concentration and species, since in a clinical setting the actual bacterial concentration in the blood culture is unknown. In the reference dataset, 94.7% of blood cultures after sample processing yielded an inoculate concentration within the stated inoculate limits of the QuickMIC system (5*10^4^ cfu/mL to 5*10^8^ cfu/mL). Notably, as blood incubation systems are highly sensitive, at positivity the bacteria have not yet reached stationary phase and will continue to grow for several hours. Thus, if a blood culture bottle turns positive during off-shift hours in a clinical situation, it could take several hours until sampling occur. To get a realistic range of starting inoculates, the blood culture bottles were started at varying timepoints during the day, using either long or short target incubation times. The time after positivity until the blood culture bottle was sampled thus ranged from 0 – 10 h, and the inoculate concentration after sample preparation ranged from 8*10^3^ to 3.7*10^7^ cfu/mL (**Figure 4**). The correlation between time after positivity and starting inoculum was investigated by linear regression, and no apparent trend with regard to species could be seen (**Figure 4A**). The general regression coefficient was determined to be close to zero (slope 0.075 log(cfu/mL)/h, 95% confidence interval 0.06 – 0.08, **Figure 4B**).

### Parameters Affecting the Performance of QuickMIC

**Figure 5:**
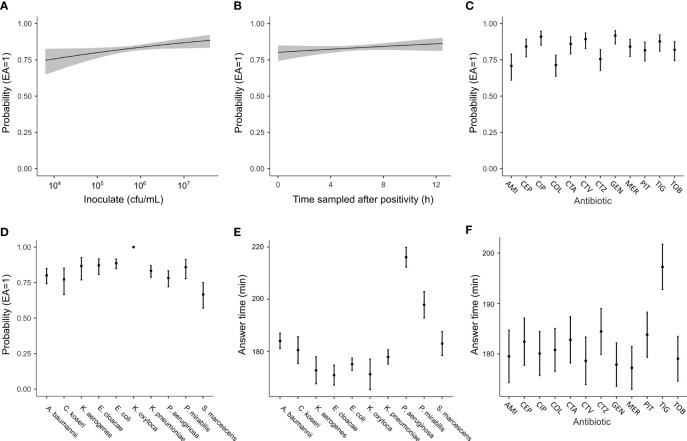
Analyses of parameters that can affect the performance of the QuickMIC test using logistic regression. The probability to achieve a result within EA as compared to the standard BMD method when analyzing spiked blood cultures is shown, and plotted against initial inoculate bacteria concentration **(A)**, the amount of time elapsed after initial indication of blood culture bottle positivity and sampling **(B)** or with regard to type of antibiotic **(C)** or bacterial species **(D)**. **(E, F)** show the estimated marginal means for the answering times grouped by either antibiotic or species, respectively. Grey fields (in **A, B**) and error bars **(C–F)** indicate 95% confidence interval in the estimated marginal means.

Rapid AST methods such as the QuickMIC method must support variations in sample properties, since the start material is unprocessed blood culture material. Parameters that can be assumed to affect the analysis are total incubation time of the blood culture (i.e. incubation time plus waiting time until the blood culture bottle is sampled), bacterial species, composition of the blood, and actual inoculate concentration. Also, the specific antibiotic used and the level of antibiotic susceptibility can be important determinants, where some antibiotics and resistance mechanisms are more difficult to measure rapidly. To investigate some of these parameters, logistic regression was performed on the spiked blood sample dataset with overall EA as dependent variable, and species, resistance category, type of antibiotic, inoculate concentration and time of sampling after positivity used as factors. The results of the logistic regression analysis are presented in **Table 5**. As expected, the type of antibiotic as well as bacterial species and resistance category were significant factors influencing the performance of the method (**Table 5**). Furthermore, initial inoculate concentration was a significant factor (p = 0.014), where low initial inoculates were correlated with lower EA (**Figure 5**). However, time of sampling after blood culture bottle positivity was did not have a significant correlation with EA (p = 0.348).

**Table 5:** Logistic regression model of important parameters affecting EA.

Predictor	χ²	Degrees of freedom (df)	p-value
Inoculum in cassette (cfu/mL)	6.060	1	0.014*
SIR category	16.598	3	<.001*
Blood culture incubation time after positivity	0.881	1	0.348
Species	59.433	9	<.001*
Antibiotic	46.597	11	<.001*

*p < 0.05.

### Performance With Clinical Samples Directly in a Hospital Microbiology Lab Setting

**Figure 6:**
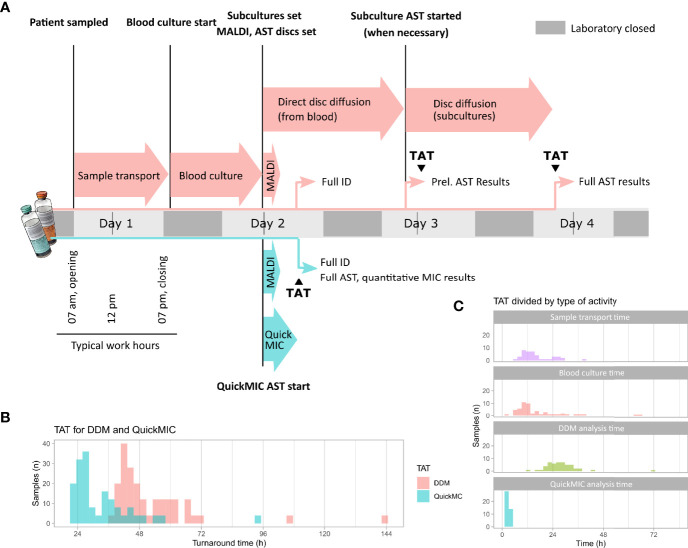
**(A)** Overview of the analyses of total turnaround time (TAT) to compare the QuickMIC method with the disc diffusion method (DDM). After patient sampling and sample transport, blood cultures were started. After positivity and during working hours, confirmed Gram-negative samples were run on QuickMIC as well as by the routine laboratory standard process (Maldi-TOF, rapid DDM followed by DDM from isolated colonies). **(B)** The difference in TAT between QuickMIC and DDM. **(C)** Breakdown of the TAT into the time periods spent during sample transport, blood culture incubation, and finally time for MIC analyses using either DDM or QuickMIC.

**Figure 7:**
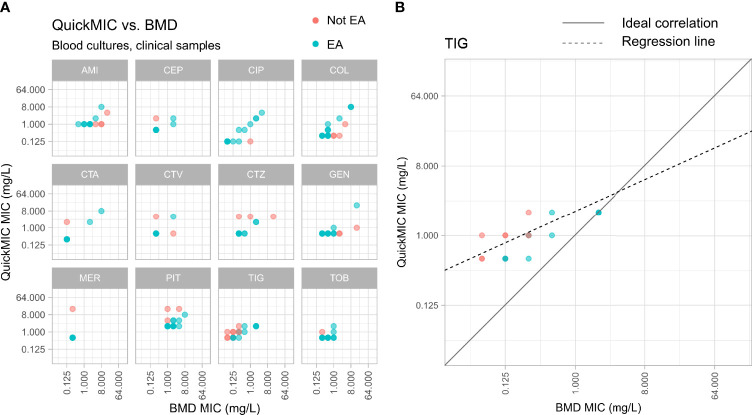
**(A)** Distribution of MIC value results from the reference BMD method (x-axis) and the QuickMIC method (y-axis), from testing of bacteria in clinical blood culture samples in response to the 12 tested antibiotics. MIC-values obtained by the QuickMIC method that are within EA in comparison to results from BMD assays are shown in blue, and MIC values not in agreement between the two methods are shown in red. **(B)** Enlarged panel showing results obtained for tigecycline (TIG). Here, a tendency for that QuickMIC in this case produces higher MIC values than the BMD reference method can be seen. The density of datapoints is represented by color gradient.

To test the capability of the QuickMIC method in a clinical setting, clinical blood culture samples (*n* = 48) were obtained from the Department of Clinical Microbiology at the Uppsala University Hospital. It was possible to analyze 41 samples (85% of total; monomicrobial, nonfastidious Gram-negative bacteria) using the QuickMIC method. The results showed that QuickMIC has 96.8% CA with DDM, as performed by the clinical laboratory (**Table 6**). The mean time to result for each drug and bacteria combination was 178 min (SD: ± 22.5 min). The samples were collected during early morning and run in the afternoon on the same day. To investigate the impact on total turnaround-time for the QuickMIC method as compared to the clinically used routine method, the time from patient sampling until test result availability (total turnaround time, TAT) was recorded for QuickMIC and compared with TAT values for DDM as per data from the clinical laboratory (**Figure 6**). In total, the mean TAT was reduced by 40%, and determined to be 33.4 h (SD: 13.0 h) for QuickMIC as compared to a TAT of 55.4 h (SD: 25.2 h) for DDM. The fastest recorded QuickMIC TAT was 21.3 h, while the fastest recorded DDM TAT was 36.1h. The QuickMIC TAT was broken up into three sub-categories, namely “transport”, “blood culture”, and “analysis” times. The transport time accounted for 48.7% (SD: 15.2%) of TAT, whereas blood culture time (the time from blood culture start to a positive signal for growth) corresponded to 41.3% (SD: 15.3%) of the TAT, and finally the time for QuickMIC analysis corresponded to 10.0% (SD: 3.0%) of the TAT. Furthermore, the collected blood culture isolates were tested with BMD for comparison (**Figure 7A**), and the degree of EA between results obtained using the QuickMIC method and BMD ranged from 45.8 – 100% (**Table 7A**, TIG and CTA, respectively). The average EA was 91.0% for all samples, and 94.2% when excluding tests with TIG. The CA between results obtained using QuickMIC and BMD ranged from 78.6 – 100% (TIG and CTA, CTV, TOB respectively). The average CA was 96.7%, and 97.6% when excluding TIG. EA and CA was significantly lower for non-fermenters (*P. aeruginosa*) as compared to Enterobacterales (**Table 7B**). There was no particular trend to the categorical errors, with MiD, MD and VMD rates of 1.0 – 1.3%. AMI, COL and GEN displayed VMD rates over 3%, however.

**Table 6:** Categorical agreement between QuickMIC and the disc diffusion method in a clinical setting.

Antibiotic *n* =	AMI (22)	CIP (37)	CTA (35)	CTZ (38)	GEN (39)	MER (36)	PIT (37)	TOB (32)	Total (276)
Categorical agreement (%)	90.9	97.0	100	97.1	97.1	97.4	94.9	94.9	96.8
MiD (%)	0.0%	3.0%	0.0%	0.0%	0.0%	0.0%	2.6%	2.6%	1.1
MD (%)	0.0%	0.0%	0.0%	0.0%	0.0%	2.6%	0.0%	2.6%	0.7
VMD (%)	9.1%	0.0%	0.0%	2.9%	2.9%	0.0%	2.6%	0.0%	1.4

**Table 7A:** Overall essential agreement and categorical agreement between QuickMIC and BMD AST of bacteria in clinical blood cultures, by tested antibiotic.

*n* =	Antibiotic												
	AMI (22)	CEP (28)	CIP (37)	COL (38)	CTA (35)	CTV (32)	CTZ (38)	GEN (39)	MER (36)	PIT (37)	TIG (33)	TOB (32)	Total (407)
EA (%)	77.8	96.4	97.2	87.9	100.0	96.7	94.4	91.4	97.1	93.5	45.8	96.3	91.0
CA (%)	92.9	96.2	96.9	96.6	100.0	100.0	96.8	96.3	96.7	100.0	78.6	100.0	96.7
MiD (%)	0.0	3.8	3.1	0.0	0.0	0.0	3.2	0.0	0.0	0.0	0.0	0.0	1.0
MD (%)	0.0	0.0	0.0	0.0	0.0	0.0	0.0	0.0	3.3	0.0	21.4	0.0	1.3
VMD (%)	7.1	0.0	0.0	3.4	0.0	0.0	0.0	3.7	0.0	0.0	0.0	0.0	1.0

**Table 7B:** Overall essential agreement and categorical agreement between QuickMIC and BMD AST of bacteria in clinical blood cultures, by tested species.

n =	Species											
	*C. freundii* (10)	*E. cloacae* (10)	*E. coli* (201)	*K. oxytoca* (52)	*K. pneumoniae* (38)	*K. variicola* (35)	*P. aeruginosa* (33)	*P. mirabilis* (10)	*P. vulgaris* (11)		Total (fermenters)(374)	Total (non-fermenters)(33)
EA (%)	80.0	87.5	91.3	90.9	93.9	91.2	68.2	88.9	90.9		91.0	68.2
CA (%)	80.0	100	95.0	100	97.5	100	82.4	77.8	90.0		95.5	82.4

## Discussion

This study demonstrates that the QuickMIC system very rapidly can provide AST data for up to 12 antibiotics against at least 10 different species of Gram-negative bacteria. The average time to result was in line with our previous study (Wistrand-Yuen et al., 2020) with a mean time of ~3 hours until result, which is significantly shorter than traditional methods such as BMD and DDM. The analysis time of the QuickMIC system is faster than the recently introduced EUCAST rapid disc diffusion (with read-outs at 4, 6 and 8 hours for select species and antibiotics), and in addition the data resolution is higher and more akin to standard DDM, which we believe is the method that is relevant for comparison. The present study further indicates that applying the QuickMIC assay can shorten turnaround times by at least 40% compared to disc diffusion. In addition, while the manual nature of the disc diffusion read-out constrains read-out times to when a trained microbiologist is available, the automated QuickMIC system could report at any time during the day. Finally, the QuickMIC system generates high-resolution quantitative MIC values, whereas disc diffusion provides qualitative susceptibility categories.

One limitation of the QuickMIC method is the relatively narrow range of the linear antibiotic concentration interval provided by the generated antibiotic concentration gradients, which is a trade-off by design to increase the assay resolution. As a result, very sensitive or very resistant strains will be reported as below or above the quantification limit and not get an on-scale MIC value. We argue that the increased resolution, leading to an increased repeatability as compared with BMD and other log2-based endpoint assays, may allow more accurate results for challenging strains with susceptibilities close to the breakpoints. A potential downside is in situations where clinical breakpoints differ by a large degree between species, which may lead to off-scale breakpoints for a specific panel of antibiotics. Since this method is supposed to be started when there is information about Gram-status, but before species identity is available, it is important to ensure that the range of drug concentrations is wide enough to provide AST results for a wide spectrum of likely pathogens. In cases where breakpoints differ greatly between species, one solution would be to generate multiple gradients spanning complementary concentration intervals of a certain antibiotic in the QuickMIC cassette. In the currently tested Gram-negative panel we do not believe this to be necessary, as all breakpoints for the here tested species are on-scale.

The performance of rapid AST methods is typically determined by either biological factors (such as state of the bacterial culture, innate bacterial growth rate, kinetics of the antibiotic effect) or technical factors (sensitivity and resolution of the system used to measure inhibition). The biological factors can to a certain extent be “optimized”; the culture can be sampled “ready to go” in an actively growing state, culture media can be optimized for growth, while the kinetics of antibiotic effects on the other hand cannot be influenced. As for the technical factors, increasing sensitivity and resolution of the system can only improve results up until hitting the limits on time imposed by the biological factors, as measuring with higher sensitivity (or much faster) will not help if the phenotypic response to the antibiotic is delayed; and slower-growing bacteria will need relatively longer measurement times, regardless of the technology used to measure growth. At the same time, a robust system must be able to handle a wide variety of starting material. It is not practical to mandate from the user to sample the culture at a very specific growth state or culture concentration. We believe the here presented QuickMIC system in principle represents the fastest possible (i.e. that we have reached the biologically imposed limits) for Gram-negative phenotypic AST under the conditions found in routine clinical microbiology; namely to support a wide range of starting inoculates (from 10^5^ to 10^8^ cfu/mL), growth states (from exponential to stationary phase after multiple hours incubation beyond positive signal from the blood culture system), growth rates (from *E. coli* to *P. aeruginosa*) and several routinely used antibiotics (from early-acting bactericidal antibiotics such as colistin to delayed-effect bacteriostatic antibiotics such as amikacin).

It is also important to note that the main goal when validating new, rapid AST methods is typically to accurately predict the antibiotic susceptibility as measured by a reference method. It is widely known that early antibiotic effects observed in time-kill assays poorly reflect the late effects. Therefore, a measured rapid “MIC_2h_” is likely to differ from a much later “MIC_18-24h_”, even when using the same method with similar conditions. The challenge of rapid AST is to use the observed MIC_2h_ data to infer the likely MIC_18-24h_ result. The QuickMIC method accomplishes this by quantifying the growth rates and morphological features of every individual bacterial microcolony throughout the antibiotic gradient chamber, thus providing a highly information-dense dataset of the antibiotic-bacteria interaction which maximizes the chance of predicting a correct MIC_18-24h_. Even so, early assessment of AST is not possible for certain combinations of antibiotics, bacterial species, and resistance mechanisms. This problem is shared with other rapid AST methods, and the viability of any new method will be dependent on whether the benefits outweigh the risks, such as a potential increase in false readouts. One obvious problem identified in this study is the varying performance for different drug and bacteria combinations, where especially TIG displayed very low EA and CA in both the reference strain and clinical blood culture datasets. Furthermore, several of the tested antibiotics displayed VMD rates that were higher than the accepted standard of 3% against the reference method. This is currently a limitation of the method. It is important to note however that the QuickMIC system is under development, and one goal of this study was to identify drug and bacteria combinations that are challenging to analyse, to guide further development. Since the performance may be affected by the read-out algorithm, or issues in the antibiotic filling process of the test cassette, or actual inherent biological differences in measuring MIC_2h_ vs. MIC_18-24h_, the performance of the system can be improved by adjusting these parameters. In **Figure 7B** it can be seen that for example the TIG MIC values as determined by the QuickMIC method are systematically higher than the reference BMD MIC values, and can therefore potentially be corrected by an adjustment of the read-out algorithm or loaded concentration of drug in the cassette.

Ultimately, the performance of a rapid AST workflow is not solely dependent on the AST method. Logistical improvements such as increased laboratory opening hours and improved sample flows are comparatively easy measures with potentially high impact in reducing sample turnaround times (Schwarzenbacher et al., 2019; Åkerlund et al., 2021). Therefore, we believe that new rapid AST methods need to simultaneously improve on assay speed but also enable usage scenarios that can help shortening sample transport and waiting times. Key to this is automation, modularity, size and usability; where a small and automated system potentially could be located nearer to the patient. It is clear from existing data that one of the main challenges in rapid diagnostics is the logistics of sample handling and opening hours of the laboratory. This is evident in the present study as well, where 48.7% of the total turnaround time was due to sample transport. To reduce the time to result in time-critical diagnostics such as in suspected blood-steam infections, hospitals have started to locate blood culturing cabinets on-site, e.g. at clinical chemistry departments who usually have around the clock opening hours, or even in satellite laboratories that are located close to the patient (Schwarzenbacher et al., 2019). This is a laudable effort, but the main problem remains that the sample once positive still must be transported to the laboratory for further AST analysis. We believe that truly rapid diagnostics will require small, modular and automated AST and ID solutions located near the blood culture cabinets, thus allowing on-site staff to start analyses around the clock, and that the here presented QuickMIC system could be part of such solutions. Incidentally, the same properties can also be argued to be beneficial when examining overall system costs for diagnostic systems in healthcare systems in low- and middle-income countries (Okeke et al., 2011; World Health Organization, 2019). In such settings, automated analysis systems with a relatively high cost per sample but low overall system costs could be an economically sound alternative to high fixed-cost centralized laboratories.

In summary, we conclude that the QuickMIC system can provide very rapid antibiotic susceptibility results of up to twelve antibiotics per test, directly from positive blood culture samples of commonly encountered Gram-negative pathogens. The method is accurate, precise and has properties which could allow for shorter sample transport chains. By providing rapid AST results, the assay could allow an earlier switch to appropriate targeted therapy, thereby enhancing the chances of survival in critically ill patients and reducing unnecessary use of broad-spectrum antibiotics. However, further improvement of the accuracy of the QuickMIC assay for specific drug and species combinations is needed.

## Data Availability Statement

The raw data supporting the conclusions of this article will be made available by the authors, without undue reservation.

## Ethics Statement

The studies involving human participants were reviewed and approved by the Swedish Ethical Review Authority. Written informed consent for participation was not required for this study in accordance with the national legislation and the institutional requirements.

## Author Contributions

Author contributions (CRedIT categories) follow: Conceptualization, CM, CJ, TT, and JK. Data curation, CM, HÖ, and JÅ. Formal analysis, CM. Funding acquisition, TT and JK. Investigation, JT, JF, CJ, and CM. Methodology, CJ and CM. Project administration, CJ and CM. Resources, TT and JK. Software, HÖ and JÅ. Supervision, TT and JK. Validation, CM and CJ. Visualization, CM. Writing – original draft, CM. Writing – review and editing, CM, CJ, TT, and JK. All authors contributed to the article and approved the submitted version.

## Funding

This study was funded by a grant to JK from Uppsala Antibiotic Centre, and by funding from Uppsala University. Reagents and consumables were kindly provided by Gradientech AB.

## Conflict of Interest

CM is a part-time employee of Gradientech AB. JT, JF, HÖ, CJ and JÅ are employees of Gradientech AB. JK is not employed by Gradientech AB but is a co-founder of Gradientech AB and owns stock in the company.

The remaining author declares that the research was conducted in the absence of any commercial or financial relationships that could be construed as a potential conflict of interest.

## Publisher’s Note

All claims expressed in this article are solely those of the authors and do not necessarily represent those of their affiliated organizations, or those of the publisher, the editors and the reviewers. Any product that may be evaluated in this article, or claim that may be made by its manufacturer, is not guaranteed or endorsed by the publisher.
